# Relationship between remission, disease activity and patient-reported outcome measures in patients with recent-onset systemic lupus erythematosus

**DOI:** 10.1177/0961203320912338

**Published:** 2020-03-18

**Authors:** Rebecca Heijke, Mathilda Björk, Martina Frodlund, Laura McDonald, Evo Alemao, Christopher Sjöwall

**Affiliations:** 1 Division of Neuro and Inflammation Sciences, Department of Clinical and Experimental Medicine, Linköping University, Linköping, Sweden; 2 Division of Occupational Therapy, Department of Social and Welfare Studies, Linköping University, Linköping, Sweden; 3 Centre for Observational Research and Data Sciences, Bristol-Myers Squibb, Uxbridge, UK; 4 World Wide Health Economics and Outcomes Research, Bristol-Myers Squibb, Princeton, NJ, USA

**Keywords:** Disease activity, EuroQoL-5 Dimensions, patient-reported outcome measures, remission, SLEDAI-2K, systemic lupus erythematosus

## Abstract

**Objective:**

Definitions of remission in systemic lupus erythematosus (SLE; DORIS (1A/1B/2A/2B)), disease activity assessments and patient-reported outcome measures (PROMs) are useful in shared decision making between patients with SLE and physicians. We used longitudinal registry data from well-characterized Swedish patients with recent-onset SLE to explore potential correlations between DORIS status or disease activity, and PROMs.

**Methods:**

Patients from the Clinical Lupus Register in North-Eastern Gothia, Sweden, who fulfilled the 1982 American College of Rheumatology and/or the 2012 Systemic Lupus International Collaborating Clinics classification criteria without prior organ damage, were enrolled at diagnosis. Data on treatments, serology, remission status (DORIS), disease activity (SLE Disease Activity Index-2000 (SLEDAI-2K)) and PROMs (quality of life: EuroQoL-5 Dimensions (EQ-5D); pain intensity, fatigue and well-being: visual analog scale (VAS) 0–100 mm) were collected during rheumatology clinic visits at months 0 (diagnosis), 6, 12, 24, 36, 48 and 60. Correlations were assessed using Pearson correlation and/or beta regression coefficients.

**Results:**

A total of 41 patients were enrolled (median age = 39 years, 80% female, 85% white). Achievement of DORIS 1A and 2A (neither of which includes serology) significantly correlated with all PROMs (EQ-5D: *p* ≤ 0.02; pain: *p* = 0.0001; fatigue: *p* = 0.0051; well-being: *p* < 0.0001). Disease activity measures were correlated with VAS pain intensity (*p* < 0.03) and VAS well-being (*p* < 0.04).

**Conclusions:**

Our findings illustrate the importance of the interplay between remission, disease activity assessments and PROMs. PROMs may be a useful tool in clinical practice, being administered prior to patient visits to streamline clinical care.

## Introduction

Systemic lupus erythematosus (SLE) is a chronic autoimmune disease that affects multiple organs and can result in diverse symptoms, including arthralgia, skin rash and fatigue.^1,2^ SLE has a significant impact on patients’ daily functioning and requires prolonged care.^2^ A systematic review of 21 randomized controlled trials and observational studies in chronic diseases demonstrated that effective communication and shared decision making between patients and physicians could improve patient health outcomes.^3^ As such, a shared decision-making strategy could also be of particular interest in SLE.

Patient-reported outcome measures (PROMs) provide information on disease activity, health-related variables and treatment from the patient’s perspective. In addition to having significantly lower health-related quality of life (HRQoL) than the general population, patients with SLE commonly report symptoms such as pain and fatigue, which, alongside well-being,^4^ are measured using the validated visual analog scale (VAS). PROMs are routinely used in clinical practice and trials to assess outcomes relevant to patients and to offer a wider perspective regarding clinical disease progression and the benefits of interventions. EuroQoL-5 Dimensions (EQ-5D) is an established generic HRQoL measure validated in rheumatoid arthritis and SLE; EQ-5D measures health status on five dimensions: mobility, self-care, usual activities, pain/discomfort and anxiety/depression.^5,6^ Combining PROMs with clinical measures provides a broader overall evaluation of the patient’s disease state. Such an approach may facilitate effective treatment communication and shared decision making between patients with SLE and their health-care providers.^1^

PROMs complement traditional assessments and are key components in shared decision making.^7^ Although remission is an important goal when employing a treat-to-target strategy in the management of SLE, there is no universally accepted definition for remission.^8^ An international task force of specialists and patient representatives recently proposed four preliminary definitions of remission in SLE (DORIS) based on established disease activity assessments (e.g. SLE Disease Activity Index (SLEDAI), Physician Global Assessment, British Isles Lupus Assessment Group (BILAG) Index), serology and ongoing treatment.^9^ For the treatment component, distinction is made between remission off and on therapy.^9^

SLE disease activity is commonly estimated using the SLEDAI-2000 (SLEDAI-2K)^10^ and/or BILAG-2004.^11^ However, SLEDAI-2K, BILAG-2004 and DORIS do not include PROMs and consequently do not provide information on patients’ perspective of their health. Due to the novelty of DORIS, limited data are available to support its performance or to show its correlation with established outcome measures.^9^

The current study used longitudinal registry data from well-characterized Swedish patients with recent-onset SLE to investigate potential correlations between DORIS status and PROMs (primary objective) and between disease activity assessments and PROMs (secondary objective).

## Methods

### Data source, patients and study design

This 60-month, retrospective, observational study used incident cases from the Clinical Lupus Register in North-Eastern Gothia, Sweden (KLURING) at the University Hospital of Linköping. The KLURING database has been described previously,^2^ and contains demographic information, including date of birth, sex, treatments, clinical outcomes and PROMs.

Consecutive, newly diagnosed adults with SLE from the KLURING database who met the 1982 American College of Rheumatology (ACR) and/or the 2012 Systemic Lupus International Collaborating Clinics classification criteria for SLE^12^ and had no prior organ damage were included. Patients were enrolled between March 2010 and October 2015 at the time of SLE diagnosis and, as in clinical practice, were seen by a rheumatologist at approximately months 0 (index date/time point of diagnosis), 6, 12, 24, 36, 48 and 60 after enrolment into the study. The protocol allowed flexibility of ±3 months per visit.

### Study assessments

Disease activity assessments included SLEDAI-2K,^10^ BILAG-2004^11^ and the number of patients meeting DORIS.^9^ At each follow-up visit, disease activity assessments, damage accrual, serology and treatment received were collected to calculate the number of patients meeting each of the remission definitions (Table 1). Patients could be on or off treatment when reaching each definition. The PROMs were: HRQoL captured using the EQ-5D-3L index^5^ and pain intensity, fatigue and general well-being, all measured using VAS 0–100 mm.

**Table 1.** Definitions of remission in SLE (DORIS).

Definition	1A	1B	2A	2B
cSLEDAI-2K = 0	✓	✓	–	–
PhGA <0.5	✓	✓	✓	✓
Serology (normal status)	–	✓	–	✓
BILAG-2004 D/E only	–	–	✓	✓

Criteria for each definition were marked with ✓ if required or – if not; patients with normal serology status have negative anti-dsDNA antibodies and normal complement blood levels.

BILAG-2004: British Isles Lupus Assessment Group 2004; cSLEDAI-2K: clinical SLE Disease Activity Index-2000; DORIS: definitions of remission in SLE; PhGA: Physician Global Assessment; SLE: systemic lupus erythematosus.

### Statistical analyses

Descriptive statistics were used to report baseline characteristics and, for DORIS status, EQ-5D, SLEDAI-2K, pain, fatigue and well-being at each time point. Pearson’s correlations were used to explore the relationship between DORIS remission and a global measure of HRQoL (EQ-5D index) and between SLEDAI-2K score and the other PROMs. Univariate mixed model analysis was used to study the relationship between pain, fatigue, well-being and DORIS. Multivariate logistic regression analysis was used to explore the relationship between DORIS and EQ-5D further, adjusting for treatment, demographic (age, sex) and clinical (body mass index, tobacco smoking, number of 1982 ACR criteria fulfilled) characteristics. Potential correlations between DORIS status and EQ-5D index were characterized based on the beta regression coefficients with *p*-values.

### Ethics approval

Oral and written informed consent was obtained from all patients. The study protocol was approved by the regional ethics review board in Linköping (M75-08/2008).

## Results

### Patients and baseline characteristics

A total of 20 (48.8%) patients had data available up to 60 months. A detailed description of the 41 patients for whom any data are available is given in Supplemental Table 1.

#### Remission and PROMs

**Figure 1.**
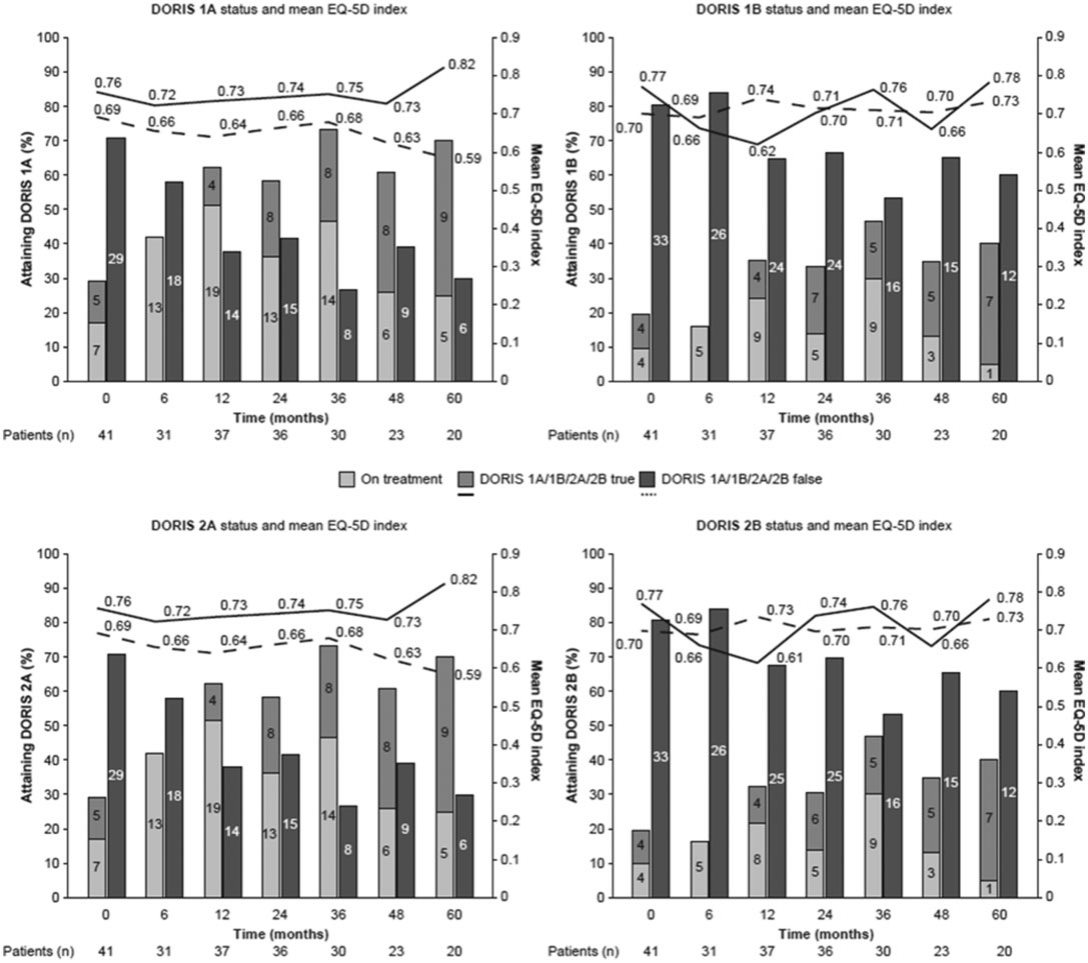
DORIS status and mean EQ-5D index over time. Bars represent the number of patients meeting (light and mid-grey) versus not meeting (dark grey) each definition of remission; light grey bars represent the number of patients on treatment among those in remission; lines represent mean EQ-5D (health-related quality of life) indices in patients meeting (solid line) versus not meeting (dashed line) each definition of remission at 0, 6, 12, 24, 36, 48 and 60 months since SLE diagnosis. DORIS: definitions of remission in SLE; EQ-5D: EuroQoL-5 Dimensions; SLE: systemic lupus erythematosus.

At month 60, the percentages of patients achieving remission were 70% (14/20) for DORIS 1A and 2A, and 40% (8/20) for DORIS 1B and 2B (Figure 1). In patients who met DORIS 1A, 1B, 2A and 2B at month 60, the mean (standard deviation (*SD*)) EQ-5D indices were 0.82 (0.11), 0.78 (0.34), 0.82 (0.11) and 0.78 (0.34), respectively. In patients who did not meet DORIS 1A, 1B, 2A and 2B at month 60, the mean (*SD*) EQ-5D indices were 0.59 (0.41), 0.73 (0.33), 0.59 (0.41) and 0.73 (0.33), respectively. Univariate logistic regression analysis showed that patients with SLE achieving remission (DORIS 1A or 2A) had higher mean EQ-5D indices compared to those not achieving remission (*p* = 0.01; regression coefficient = 0.09; Table 2). This relationship remained significant after adjusting for baseline covariates (*p* = 0.02; regression coefficient = 0.08). No significant relationship was observed between remission (DORIS 1B or 2B) and EQ-5D indices over the study period. Beta regression coefficients generated using univariate mixed model analysis demonstrated that patients who achieved remission had, on average, more favourable fatigue scores (DORIS 1A and 2A: *p* < 0.01; DORIS 1B: *p* = 0.03; DORIS 2B: *p* = 0.02), well-being scores (DORIS 1A, 1B, 2A and 2B: *p* < 0.01) and pain scores (DORIS 1A and 2A: *p* < 0.01; DORIS 1B: *p* = 0.02; DORIS 2B: *p* = 0.01) compared to those not achieving remission (Table 2).

**Table 2.** Beta regression coefficients for DORIS status and PROMs.

	EQ-5D				VAS pain		VAS fatigue		VAS well-being	
	Without adjustment		With adjustment^a^		Without adjustment		Without adjustment		Without adjustment	
	β regression coefficient	*p*-Value	β regression coefficient	*p*-Value	β regression coefficient	*p*-Value	β regression coefficient	*p*-Value	β regression coefficient	*p*-Value
DORIS 1A	0.09	0.01	0.08	0.02	−13.13	<0.01	−11.62	0.01	−18.16	<0.01
DORIS 1B	<−0.01	0.93	−0.02	0.66	−8.18	0.02	−10.05	0.03	−11.26	<0.01
DORIS 2A	0.09	0.01	0.08	0.02	−13.13	<0.01	−11.62	0.01	−18.16	<0.01
DORIS 2B	0.01	0.86	−0.01	0.85	−9.03	0.01	−10.36	0.02	−12.76	0.01

Analysis included patients either on or off treatment.

^a^Adjusted for demographic (e.g. sex), clinical (e.g. BMI, tobacco smoking) and treatment covariates.

BMI: body mass index; DORIS: definitions of remission in SLE; EQ-5D: EuroQoL-5 Dimensions; PROMs: patient-reported outcome measures; SLE: systemic lupus erythematosus; VAS: visual analog scale.

### Disease activity and PROMs

Over 60 months, disease activity (SLEDAI-2K score) and PROMs (EQ-5D, pain, fatigue and well-being scores) remained mostly constant, with a slight numerical decrease in SLEDAI-2K score over time (Supplementary Figure 1). Statistically significant correlations were identified between SLEDAI-2K and pain scores at months 6, 36 and 48 (*p* < 0.01–0.03; Table 3). No significant correlations were identified between SLEDAI-2K and pain scores at other time points (*p* = 0.11–1.00). SLEDAI-2K score did not correlate significantly with EQ-5D (*p* = 0.22–0.90) or fatigue scores (*p* = 0.09–0.87) at any time point. Significant correlation of SLEDAI-2K score with well-being score was observed at month 48 (*p* = 0.03).

**Table 3.** Pearson correlations between SLEDAI-2K scores and PROMs.

Time since SLE diagnosis (months)	SLEDAI-2K and EQ-5D		SLEDAI-2K and VAS pain		SLEDAI-2K and VAS fatigue		SLEDAI-2K and VAS well-being	
	Pearson correlation coefficient	*p*-Value	Pearson correlation coefficient	*p*-Value	Pearson correlation coefficient	*p*-Value	Pearson correlation coefficient	*p*-Value
0 (*n* = 41)	0.16	0.34	−0.17	0.33	−0.35	0.09	0.15	0.38
6 (*n* = 31)	−0.23	0.21	0.42	0.02	0.18	0.39	0.34	0.07
12 (*n* = 37)	0.18	0.28	<0.01	1.00	0.15	0.41	0.10	0.55
24 (*n* = 36)	0.08	0.63	0.12	0.50	0.07	0.71	0.03	0.84
36 (*n* = 30)	−0.02	0.90	0.40	0.03	0.30	0.11	0.31	0.10
48 (*n* = 23)	−0.27	0.22	0.55	0.01	0.09	0.70	0.46	0.03
60 (*n* = 20)	−0.24	0.31	0.38	0.11	−0.04	0.87	0.42	0.08

## Discussion

Using longitudinal data from a well-documented Swedish registry, this study showed significant correlations between achievement of remission (DORIS 1A and 2A; neither of which includes serology) and HRQoL, pain, fatigue and well-being; between disease activity (SLEDAI-2K) and pain intensity at months 6, 36 and 48; and between SLEDAI-2K and well-being at month 48. This indicated that while DORIS and SLEDAI-2K are both useful variables in clinical practice, they may reflect different aspects of the disease burden of patients with SLE. No significant correlations were found between SLEDAI-2K and HRQoL or fatigue. The results of this study were largely consistent with previous studies. However, they offer a unique real-world perspective.

Limited data are available on the performance of the proposed SLE remission definitions and their relationship with other established outcome measures potentially due to the relatively recent proposal of DORIS.^9^ To evaluate the performance of DORIS, data from both randomized controlled trials and real-world studies are needed. In a post hoc analysis of data from the belimumab trials in SLE, BLISS-52 (*N* = 865) and BLISS-76 (*N* = 819), the performance of and differences between the four remission definitions were evaluated.^13^ During the follow-up in the BLISS trials, few patients achieved remission according to DORIS. Furthermore, fewer patients achieved remission based on definitions that include serology (DORIS 1B and 2B) versus those that do not (DORIS 1A and 2A).^13^ Our study complements the post hoc analysis from the BLISS trials by providing a real-world perspective and describing associations between achievement of DORIS and HRQoL. To our knowledge, this is the first study to demonstrate the real-world performance of DORIS in patients with recent-onset SLE.

In our study, SLEDAI-2K scores did not correlate with HRQoL. A systematic review of 53 studies in SLE found that disease activity did not correlate with HRQoL.^14^ The lack of association between disease activity and HRQoL may reflect that these measures capture different aspects of SLE, and that objective disease measures and PROMs may not follow the same course over time. The correlation between remission and HRQoL likely reflects a parameter included in DORIS but not SLEDAI-2K.

An association between disease activity and pain intensity was seen. Similarly, in a study of 60 paediatric patients with SLE, VAS pain score showed weak to moderate positive correlations with disease activity.^4^ Interestingly, no disease activity assessment showed significant correlations with eight other HRQoL measures or psychological variables, including fatigue, anxiety, mood and sleep.^4^ We also found that disease activity did not correlate significantly with fatigue. A systematic review of 34 studies in patients with SLE also reported that SLEDAI score did not correlate with fatigue. However, a correlation between disease activity, assessed using the Systemic Lupus Activity Measure (excluding the fatigue domain), was observed.^15^

The significant correlations between disease activity and well-being reported here are consistent with results from a retrospective, observational study that identified correlations between disease-specific measures and the physical component summary score of the Short-Form (36) Health Survey.^16^ Together, our findings and those from previous studies show that careful consideration should be given to the disease activity assessment tool used when interpreting any correlation with PROMs, as each measure of disease activity comprises a range of assessments. The results presented here also highlight the importance of understanding the patient experience: while objective measures may indicate good disease control, these may not reflect patients’ perceptions of their own health status. It is important for rheumatologists to distinguish symptoms related to active SLE, which require additional immunosuppression, from those that would benefit from interventions provided by a multi-professional rehabilitation team.

Limitations of the study should be considered. Compared to randomized controlled trials, real-world studies are, by nature, prone to selection bias. Although Swedish healthcare is tax funded and offers universal access, thereby limiting initial risk of selection bias, certain patients may have more missing observations than others (e.g. patients with mild SLE require fewer visits than patients with severe disease). Additionally, a multivariate analysis adjusting for demographic, clinical and treatment covariates was conducted to remove the effects of any potential bias or confounding factors in the DORIS–EQ-5D analysis. That significant correlations of disease activity with pain and well-being were identified at some, but not all, time points may be the result of the reduced statistical power of a small patient population and fewer patients remaining in the study cohort over time. Caution must be taken to avoid over-interpreting these data due to the low patient numbers. Although limited by its sample size, our study utilized data from a very well-characterized patient population which enabled evaluations of multiple outcome measures over an extended time period. It should be noted that the majority of patients in this study were white, which may hinder generalization of the findings to other ethnicities. Finally, due to the correlational nature of these results, it is not possible to determine if remission is causally implicated in improved HRQoL.

In summary, we report the first data on the real-world performance of DORIS, and have identified correlations between remission, disease activity assessments and PROMs. Further evaluation of the performance of DORIS in larger, longitudinal studies is warranted before implementation of these definitions into routine clinical practice. The correlations observed in this study highlight the importance of understanding the relationship of, and differences between, disease activity assessments and PROMs, which is key to successful outcomes in shared decision making and SLE management. These results indicate that PROMs may be a useful tool in clinical practice, being administered prior to patient visits to streamline clinical care. Correlations of a broader range of disease activity assessments with PROMs should be explored to benefit SLE management further.

## Supplemental Material

LUP912338 Supplemental Material - Supplemental material for Relationship between remission, disease activity and patient-reported outcome measures in patients with recent-onset systemic lupus erythematosus Click here for additional data file. Supplemental material, LUP912338 Supplemental Material for Relationship between remission, disease activity and patient-reported outcome measures in patients with recent-onset systemic lupus erythematosus by Rebecca Heijke, Mathilda Björk, Martina Frodlund, Laura McDonald, Evo Alemao and Christopher Sjöwall in Lupus
