# Supplementary material for: Relationship between remission, disease activity and patient-reported outcome measures in patients with recent-onset systemic lupus erythematosus
Source: Lupus. 2020 Mar 18;29(6):625–30. doi: 10.1177/0961203320912338 (PMC7184926; doi:10.1177/0961203320912338)

Supplemental material, for ‘Relationship between remission, disease activity and patient-reported outcome measures in patients with recent-onset systemic lupus erythematosus’ by Rebecca Heijke, Mathilda Björk, Martina Frodlund, Laura McDonald, Evo Alemao and Christopher Sjöwall

Definition of on/off therapy:

Off therapy: No immunomodulatory treatment other than antimalarials.

On therapy: Stable immunosuppressives, including biologics, and prednisolone up to 5 mg daily allowed.

Supplementary Table 1 Patient characteristics at baseline (unless otherwise specified)

Data are presented as n (%) unless otherwise stated.

ACR: American College of Rheumatology; IF-ANA: immunofluorescence microscopy antinuclear antibodies; SLICC: Systemic Lupus International Collaborating Clinics.

**Included patients**	**N = 41**
Background variables	
Sex, female	33 (80.5)
Age at inclusion, years, median (range)	39 (18–77)
White	35 (85.4)
Previous or current tobacco smoking	18 (43.9)
Meeting the 1982 ACR classification	36 (87.8)
Meeting the 2012 SLICC classification	41 (100.0)
Number of fulfilled ACR criteria, mean (range)	4.7 (3–9)
Meeting DORIS criteria	
1A	12 (29.3)
1B	8 (19.5)
2A	12 (29.3)
2B	8 (19.5)
SLICC/ACR damage index at last follow-up (score), mean (range)	0.49 (0–2)
No organ damage at last follow-up	25 (61.0)
Clinical phenotypes (1982 ACR definitions)	
1) Malar rash	12 (29.3)
2) Discoid rash	4 (9.8)
3) Photosensitivity	20 (48.8)
4) Oral ulcers	7 (17.1)
5) Arthritis	29 (70.7)
6) Serositis	11 (26.8)
7) Renal disorder	15 (36.6)
8) Neurologic disorder	3 (7.3)
9) Hematologic disorder	27 (65.9)
10) Immunologic disorder	24 (58.5)
11) IF-ANA	41 (100.0)
Other characteristics	
Prescribed antihypertensives	19 (46.3)
Prescribed antidepressants	4 (9.8)

Raynaud's phenomenon	11 (26.8)
Estimated glomerular filtration rate at last follow-up (mL/min/1.73 m^2), mean (range)	98.6 (29–171)

Supplementary Figure 1 Mean scores over time

Scores shown are for A) SLEDAI-2K, B) EQ-5D, C) VAS pain, D) VAS fatigue, E) VAS well-being. Data are mean (standard deviation). Pain, fatigue and well-being: VAS ranging from 0–100, with higher scores indicating greater severity.

EQ-5D: EuroQoL-5 Dimensions; SLE: systemic lupus erythematosus; SLEDAI-2K: SLE Disease Activity Index-2000; VAS: visual analogue scale.

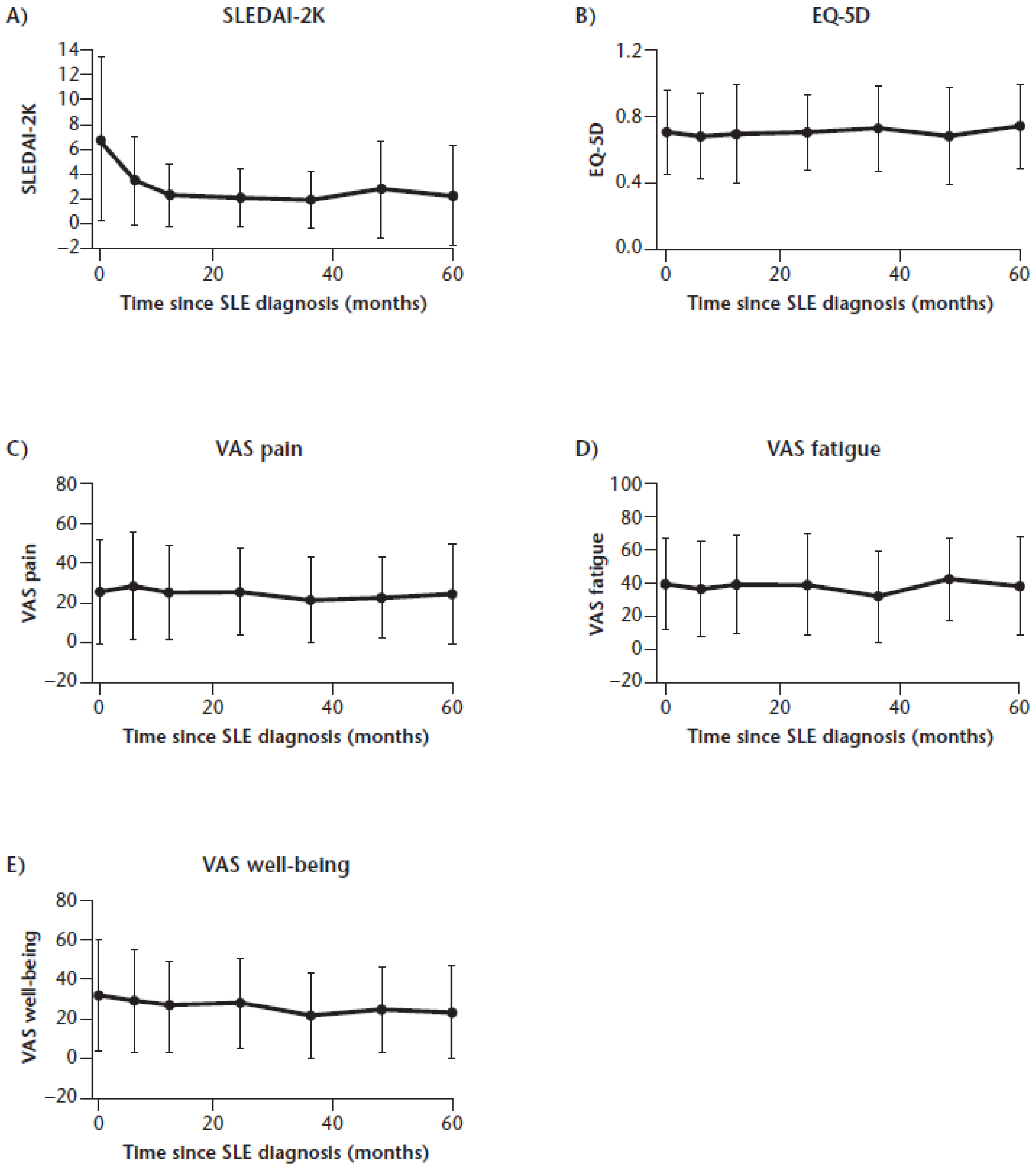

Supplement: LUP912338 Supplemental Material - Supplemental material for Relationship between remission, disease activity and patient-reported outcome measures in patients with recent-onset systemic lupus erythematosus [file LUP912338_Supplemental_Material.pdf]
